# Supplementary material for: Thinking in pictures in everyday life situations among autistic adults
Source: PLoS One. 2021 Jul 22;16(7):e0255039. doi: 10.1371/journal.pone.0255039 (PMC8297849; doi:10.1371/journal.pone.0255039)
Supplement: S1 Appendix — (DOCX) [file pone.0255039.s002.docx]

**S1 Appendix:**

**Original French version of the questionnaire**

**Les questions suivantes portent sur vos expériences internes au quotidien. Merci, si possible, de donner des détails et des précisions quand cela vous est demandé :**

Lorsque vous visualisez un objet ou une situation mentalement (image interne) :

1) Cette image interne est-elle détaillée ou floue ou parfois floue et parfois détaillée ?

2) Cette image interne est-elle persistante ou de courte durée ou parfois persistante et parfois de courte durée ?

3) Parvenez-vous à manipuler cette image interne (par exemple changer de point de vue, zoomer, etc.) ? Toujours ou jamais ou parfois ?

4) Décrivez ce qui vous vient à l'esprit lorsque vous entendez le nom d'une ville dans laquelle vous êtes déjà allé, comme *XX* *(nom d'une ville bien connue de tous les participants).*

5) Lorsque vous vous rappelez de quelque chose (que vous avez lu, vu ou entendu), avez-vous tendance à utiliser des images ou des mots ?

Mots / Images / Les deux

6) Pour comprendre ce que l’on vous explique (ex : des instructions que l’on vous donne), avez-vous tendance à utiliser des images ou des mots ?

Mots / Images / Les deux

7) Lorsque vous anticipez un événement à venir (ex : si vous devez vous rendre quelque part), avez-vous tendance à utiliser des images ou des mots ?

Mots / Images / Les deux

8) Lorsque vous planifiez des activités (ex : si vous réfléchissez à comment organiser votre journée), avez-vous tendance à utiliser des images ou des mots ?

Mots / Images / Les deux

9) Lorsque vous êtes face à un problème et que vous ne trouvez pas la solution, avez-vous tendance à utiliser des images ou des mots ?

Mots / Images / Les deux

10) Lorsque vous devez prendre une décision (ex : le meilleur chemin pour aller quelque part), avez-vous tendance à utiliser des images ou des mots ?

Mots / Images / Les deux

11) Avez-vous tendance à utiliser des images ou des mots lorsque vous devez mémoriser quelque chose (ex : une liste de courses, un numéro de téléphone, …) ?

Mots / Images / Les deux
