# Supplementary material for: Thinking in pictures in everyday life situations among autistic adults
Source: PLoS One. 2021 Jul 22;16(7):e0255039. doi: 10.1371/journal.pone.0255039 (PMC8297849; doi:10.1371/journal.pone.0255039)
Supplement: S2 Appendix — (DOCX) [file pone.0255039.s003.docx]

**S2 Appendix :**


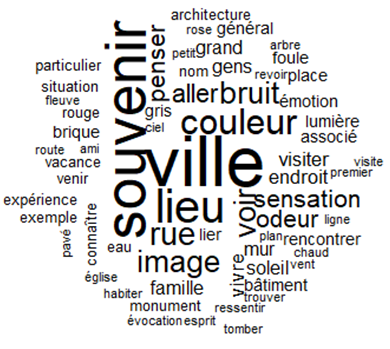


*Fig. 5. Original French word cloud for the control group.*


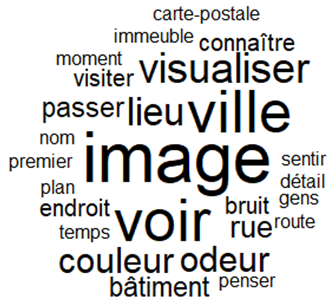


*Fig. 6. Original French word cloud for the autistic group.*
