# Supplementary material for: Thinking in pictures in everyday life situations among autistic adults
Source: PLoS One. 2021 Jul 22;16(7):e0255039. doi: 10.1371/journal.pone.0255039 (PMC8297849; doi:10.1371/journal.pone.0255039)
Supplement: S3 Appendix — (DOCX) [file pone.0255039.s004.docx]

**S3 Appendix**:


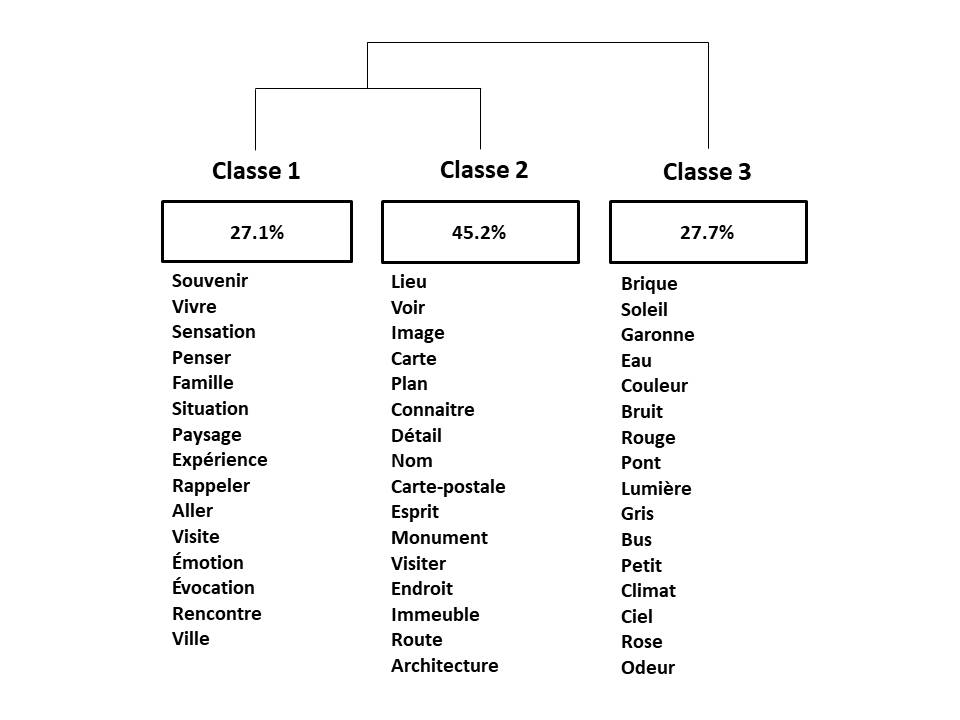


*Fig. 7. Original French dendrogram for the Descending hierarchical classification of lexical classes.*
